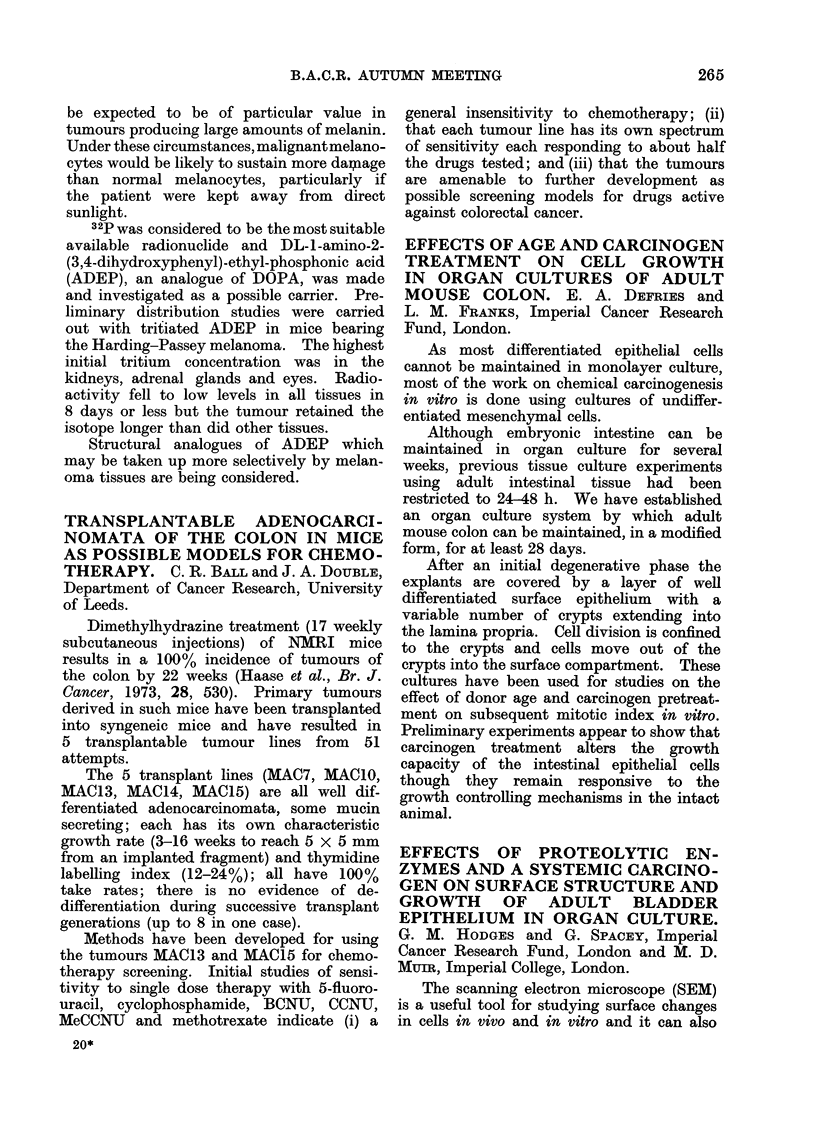# Proceedings: Effects of age and carcinogen treatment on cell growth in organ cultures of adult mouse colon.

**DOI:** 10.1038/bjc.1975.54

**Published:** 1975-02

**Authors:** E. A. Defries, L. M. Franks


### EFFECTS OF AGE AND CARCINOGEN TREATMENT ON CELL GROWTH IN ORGAN CULTURES OF ADULT MOUSE COLON.

E. A. Defries and L. M. Franks, Imperial Cancer Research Fund, London.

As most differentiated epithelial cells cannot be maintained in monolayer culture, most of the work on chemical carcinogenesis *in vitro* is done using cultures of undifferentiated mesenchymal cells.

Although embryonic intestine can be maintained in organ culture for several weeks, previous tissue culture experiments using adult intestinal tissue had been restricted to 24–48 h. We have established an organ culture system by which adult mouse colon can be maintained, in a modified form, for at least 28 days.

After an initial degenerative phase the explants are covered by a layer of well differentiated surface epithelium with a variable number of crypts extending into the lamina propria. Cell division is confined to the crypts and cells move out of the crypts into the surface compartment. These cultures have been used for studies on the effect of donor age and carcinogen pretreatment on subsequent mitotic index *in vitro*. Preliminary experiments appear to show that carcinogen treatment alters the growth capacity of the intestinal epithelial cells though they remain responsive to the growth controlling mechanisms in the intact animal.